# Optical Defocus Rapidly Changes Choroidal Thickness in Schoolchildren

**DOI:** 10.1371/journal.pone.0161535

**Published:** 2016-08-18

**Authors:** Danyang Wang, Rachel Ka Man Chun, Manli Liu, Roger Pak Kin Lee, Yuan Sun, Ting Zhang, Chuen Lam, Quan Liu, Chi Ho To

**Affiliations:** 1 State Key Laboratory of Ophthalmology, Zhongshan Ophthalmic Center, Sun Yat-sen University, Guangzhou, PR China; 2 Laboratory of Experimental Optometry, Centre for Myopia Research, School of Optometry, The Hong Kong Polytechnic University, Hong Kong, PR China; 3 Hainan Eye Hospital, Zhongshan Ophthalmic Center, Sun Yat-Sen University, Haikou, PR China; Medizinische Universitat Graz, AUSTRIA

## Abstract

The current study aimed to examine the short-term choroidal response to optical defocus in schoolchildren. Myopic schoolchildren aged 8–16 were randomly allocated to control group (CG), myopic defocus group (MDG) and hyperopic defocus group (HDG) (n = 17 per group). Children in MDG and HDG received additional +3D and -3D lenses, respectively, to their full corrections on the right eyes. Full correction was given to their left eyes, and on both eyes in the CG. Axial length (AXL) and subfoveal choroidal thickness (SFChT) were then measured by spectral domain optical coherence tomography. Children wore their group-specific correction for 2 hours after which any existing optical defocus was removed, and subjects wore full corrections for another 2 hours. Both the AXL and SFChT were recorded hourly for 4 hours. The mean refraction of all subjects was -3.41 ± 0.37D (± SEM). SFChT thinned when exposed to hyperopic defocus for 2 hours but less thinning was observed in response to myopic defocus compared to the control group (p < 0.05, two-way ANOVA). Removal of optical defocus significantly decreased SFChT in the MDG and significantly increased SFChT in the HDG after 1 and 2 hours (mean percentage change at 2-hour; control vs. hyperopic defocus vs. myopic defocus; -0.33 ± 0.59% vs. 3.04 ± 0.60% vs. -1.34 ± 0.74%, p < 0.01). Our results showed short-term exposure to myopic defocus induced relative choroidal thickening while hyperopic defocus led to choroidal thinning in children. This rapid and reversible choroidal response may be an important clinical parameter in gauging retinal response to optical defocus in human myopia.

## Introduction

The choroid is the posterior part of the uveal tract in the eye and it supplies nutrients and oxygen to the outer retina and modulates retinal temperature [1]. The choroid is involved in myopia development since it thins significantly in animal models of myopia, e.g., in chicks [2], guinea pigs [3] and monkeys [4]; conversely, it thickens substantially in hyperopic animal eyes. When the myopigenic perturbation (either form deprivation or optical defocus) is removed, the choroid will thicken again and return to baseline values [5]. This choroidal expansion was shown to occur within minutes in chicks [6]. It was hypothesized that the choroidal thickening acts to push the retinal plane forward to receive clear images when it is optically defocused [1]. In addition, the choroid may modulate its blood flow during the active eye growth. The choroidal blood flow was decreased in myopic chicks while it increased significantly in chicks undergoing recovery from myopia [7]. The increase in the blood flow was rapid and occurred before choroidal thickening during recovery. Thus, the blood flow has been proposed to contribute to the change in choroidal thickness that follows it. However, the mechanism involved in choroidal blood flow regulation and its role in ocular development remains incompletely understood. Apart from the choroidal blood flow, researchers have proposed the non-vascular smooth muscles along the choroidal vessels may contribute to the rapid change of choroidal thickness. The non-vascular smooth muscles may alter its tonus to facilitate the fluid flow across the lacunae of the choroid [8]. Choroidal permeability has been investigated most extensively in chicks because their change in choroidal thickness during ocular development is the most pronounced across a range of animals and their choroidal vessels have a lymphatic vessel-like structure. Their permeability was studied indirectly via investigating the suprachoroidal fluid which is the fluid in between the choroid and sclera. The protein concentration of the suprachoroidal fluid was significantly up-regulated during recovery from myopia [9, 10] indicating an increase in choroidal permeability. These results suggest a role for the choroid in the regulation of eye growth.

Choroidal thickness can be easily captured and measured in humans non-invasively using spectral domain optical coherence tomography (SD-OCT). The chorio-scleral interface can be clearly visualized. The thickness of choroids in young adults and children has been profiled in other studies indicating the feasibility of assessing choroidal thickness in humans via SD-OCT [11, 12]. The prevalence of myopia has reached epidemic levels in some Asian areas such as Hong Kong [13], Taiwan [14] and Singapore [15]. High myopes are more prone to certain sight threatening diseases like retinal detachment [16] and glaucoma [17]. Children who develop myopia at young age tend to have a faster rate of axial elongation [18]. A number of interventions are available to control axial elongation, and, thereby, myopic progression in children, e.g., contact lenses and spectacles inducing myopic defocus [19, 20]. Myopic defocus has been shown to slow down myopic progression in both animal models [21] and children [19]. Myopic defocus can be induced by positive powered lenses. Choroids thickened significantly in response to the myopic defocus in animals. Recently, the choroidal response to optical defocus has been reported in young adults and it was shown to thicken when treated with myopic defocus [22]. However, whether the choroid similarly responds to defocus in children, who are more vulnerable to myopic progression, remains unknown. A recent finding also demonstrated an association between choroidal thickness and eye growth in children. Children with faster axial elongation had less thickening of subfoveal choroidal thickness in a longitudinal study [23]. Therefore, the purpose of this study is to investigate the short-term choroidal response to both hyperopic and myopic defocus in schoolchildren using SD-OCT.

## Materials and Methods

### Subjects

Chinese schoolchildren aged 8 to 16 years with myopia between -0.50D and -6.00D, astigmatism of less than 1.00D, and anisometropia of less than 1.00D were enrolled in this double-masked, single-center, randomized controlled study. Subjects were required to have the monocular best corrected visual acuity of logMAR 0.0 or better. Those with a history of significant ocular or systemic diseases, ocular trauma or surgery, or a history of myopia control interventions such as orthokeratology, soft contact lens, rigid gas permeable lens wear and atropine in the past one month were excluded from this study. Written informed consent was obtained from both children and their parents. The study protocol was approved by The Ethical Review Committee of Guangzhou Zhongshan Ophthalmic Center and adhered to the tenets of the Declaration of Helsinki.

### Procedures

Cycloplegic refraction was carried out by instilling three drops of 1% cyclopentolate hydrochloride (Chauvin pharmaceuticals Ltd., London, UK) at 10-minute intervals, starting at 9:00 a.m. in order to eliminate the potential effect of accommodation on choroidal thickness. Although the recent studies only reported changes in choroidal thickness toward accommodation in young adults [24, 25], we anticipate the effect of accommodation on the choroidal thickness may be more significant because of the larger amplitude of accommodation in children. Additionally, it is difficult to control the accommodation in children by just asking them to view distant objects. Therefore, cycloplegic agents were applied to all children.

**Fig 1:**
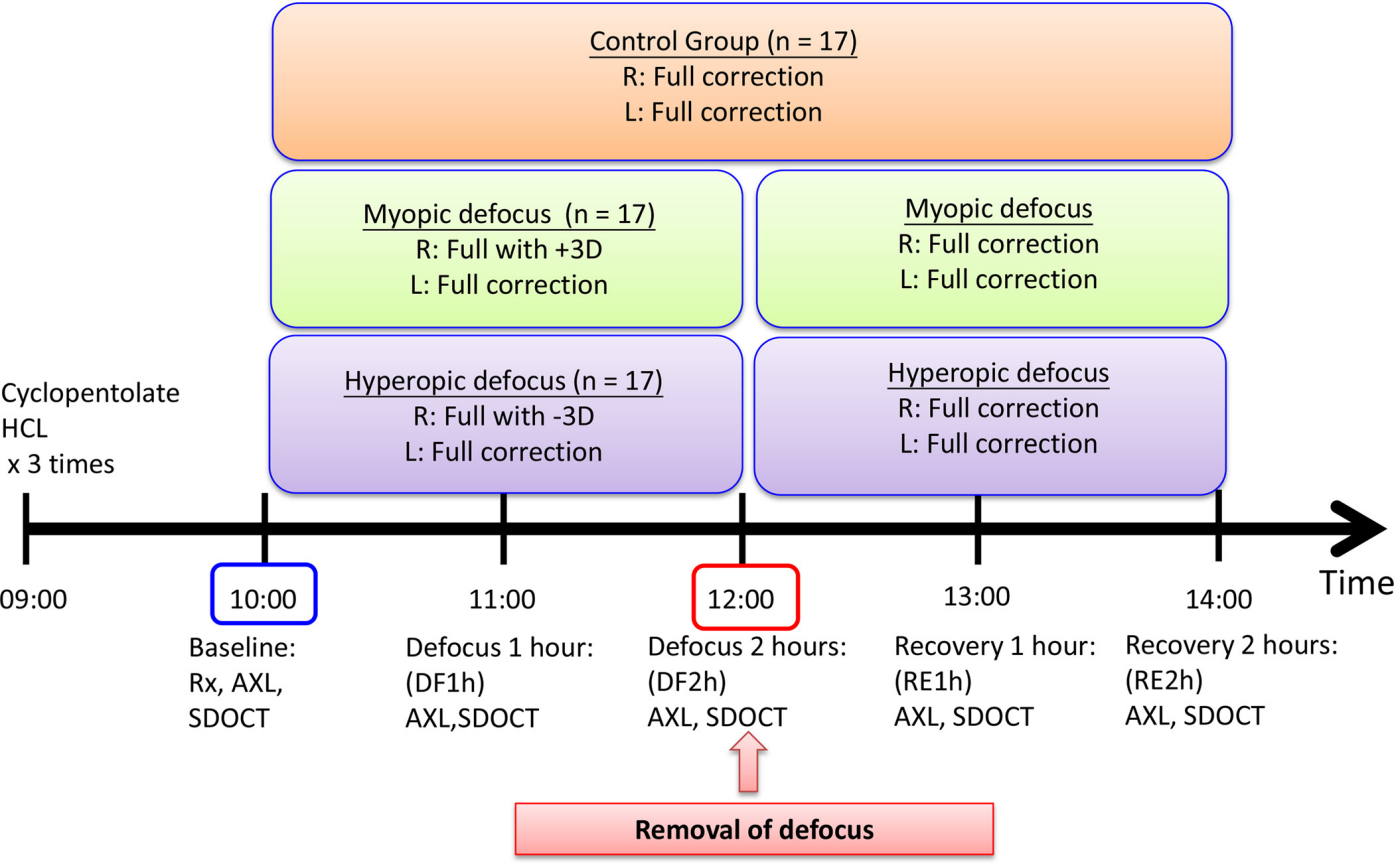
Study design. Baseline measurements including refractive errors (Rx), axial length (AXL) and OCT images were taken after cycloplegia. Subjects were randomly assigned into control, myopic defocus or hyperopic defocus group and defocus lenses were worn for 2 hours using trial lenses. Defocus lenses were then removed after 2 hours of lens wear. AXL and OCT images were acquired every hour.

After cycloplegia for at least 40 minutes, refraction in spherical equivalent (SE), axial length (baseline) and choroidal thickness (baseline) were measured. The children were then randomly allocated into three groups: control group (CG), myopic defocus group (MDG) and hyperopic defocus group (HDG) according to a random number table (Excel, Microsoft, Redmond, WA). Parents and children were kept masked to the assigned groups. Children in MDG and HDG wore +3D and -3D lenses on top of their distant refractive errors in their right eyes using trial lenses and frame, respectively, while the left eyes were fitted with fully corrected lenses for two hours. After two hours of lens wear, both eyes received full correction of distant refraction for another 2 hours (recovery period). Children in CG received full correction at distance for 4 hours as control (Fig 1). Axial length and choroidal thickness were monitored every hour for 4 hours. The children were required to watch television at a distance of 5–6 meters in a room with natural lighting. The mean room luminance was 430 lux with the range from 368 to 499 lux. All choroidal thickness and axial length data was collected at 10:00 a.m. (after an hour of cycloplegia) to avoid variation due to diurnal fluctuation.

Axial length was measured by a non-contact partial coherence interferometer, IOL-master (Carl Zeiss Meditec Inc., Dublin, CA). The axial length is defined as the distance between anterior cornea and retinal pigment epithelium (RPE). Six readings were collected and averaged. The operator was masked from the children’s group allocation. Choroidal images were captured using spectral domain optical coherence tomography (SD-OCT, Spectralis HRA+OCT, Heidelberg Engineering, Heidelberg, Germany). This device utilized a light source with a peak wavelength of 870 nm and a scanning speed of 40000 A-scan/sec to provide cross sectional retinal and choroidal images with axial resolution of 3.9 μm and transverse resolution of 14 μm. Enhanced depth image scanning mode was used during the image acquisition. At each session, three 9-mm horizontal and vertical fovea centered line scans (each comprised of 100 averaged scans) were obtained. The choroidal image from the baseline measurement was set as reference map and all the following images were captured according to the reference map to ensure the same position was being scanned afterwards in the serial hourly scans.

In order to minimize the errors induced by taking off the lenses during the data measurement, IOL-master and SD-OCT were placed in the same room and close to the area where children stayed.

### Choroidal thickness measurement

**Fig 2:**
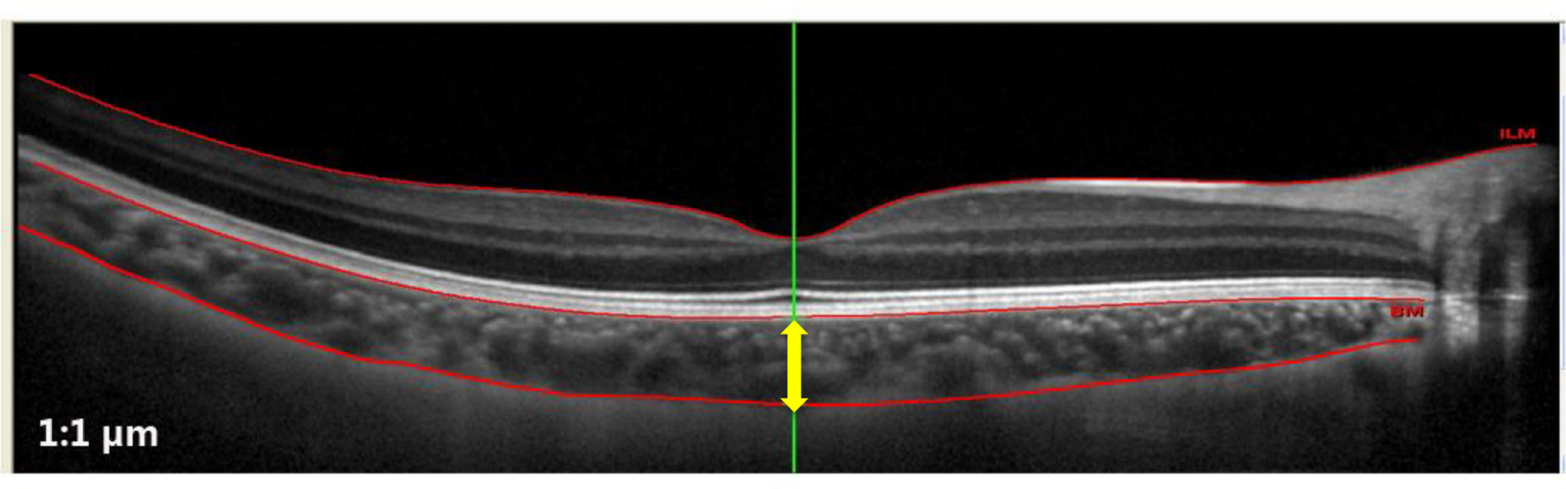
A representative OCT image showing choroid using enhanced depth imaging (EDI) mode. Subfoveal choroidal thickness was determined manually by two independent experienced observers using built-in software.

Subfoveal choroidal thickness (SFChT) was manually measured by two experienced and masked observers using built-in Heidelberg eye explorer software (version 5.8.3.0, Heidelberg Engineering, Heidelberg, Germany). The thickness of choroid was measured from the outer portion of the hyper-reflective line corresponding to the retinal pigmented epithelium to the inner surface of the sclera (Fig 2). A total of 6 measurements for SFChT were made from three horizontal and three vertical OCT images at each time point. All measurements were performed in 1:1 μm viewing mode and two times magnification was used during choroidal thickness measurement.

### Statistical analysis

Statistical analysis was performed using statistical software (SPSS version 18.0; SPSS Inc., Chicago, IL). All data are presented as mean ± standard error of the mean (SEM). Interobserver reproducibility between choroidal thicknesses measured by two observers was evaluated based on the intraclass correlation coefficient (ICC). Baseline data were analyzed using one-way ANOVA between different groups. Data of the right and left eye were analyzed separately in order to test for crossover effects. Regarding the axial length and choroidal thickness, general linear model-repeated measurement was used to investigate the changes within groups. Two-way ANOVA was used to compare proportional changes between control, myopic defocus and hyperopic defocus groups (with time and group as within-subject factor and between-subjects factor, respectively), and between right and left eyes in each group (with time and eye as within-subjects factor and between-subjects factor, respectively).

In the first 2 hours of the experiment, the changes in axial length and choroidal thickness were compared to the baseline measurement. After 2 hours of treatment, all children received full correction at distance as recovery period. The changes were then compared to the measurement after 2 hours of defocus lens wear.

The percentage change in AXL or choroidal thickness in the first two hours was calculated as the proportional difference between baseline and data at 1-hour or 2-hours. The percentage change in AXL and choroidal thickness during the recovery period in the last 2 hours was calculated as the percentage difference between 2-hour data and data at 3-hour or 4-hours. Fisher’s least significant difference test was used for post-hoc analysis. A p-value below 0.05 was considered statistically significant.

## Results

Fifty one children participated in this study (n = 17 per group, 3 groups in total). Their baseline data are shown in Table 1. All children involved were myopic with the refractive errors ranging from -1.50D to -6.63D (-3.41 ± 0.37D; mean ± SEM) in terms of spherical equivalent. There was no significant difference in the baseline data between groups (one-way ANOVA, p > 0.05). All OCT images were analyzed by two independent observers and the inter-examiner reproducibility of choroidal thickness measurement was assessed. The ICC for the choroidal thickness measurement was 0.988 which indicated a high reproducibility among the observers.

**Table 1:** Baseline data (mean ± SEM) in control, myopic defocus and hyperopic defocus group.

	Control	Myopic Defocus	Hyperopic Defocus	p-value
**Age / Years**	12.06 ± 0.49	12.29 ± 0.58	12.18 ± 0.53	0.953
**Rx / D**	-3.31 ± 0.32	-3.67 ± 0.45	-3.26 ± 0.34	0.702
**Axial length / mm**	25.02 ± 0.28	25.24 ± 0.21	25.87 ± 0.19	0.528
**SFChT / μm**	255.29 ± 11.74	241.40 ± 15.18	225.87 ± 15.26	0.347

Rx denotes refractive error in spherical equivalent while SFChT represents the subfoveal choroidal thickness. P-value was calculated by one-way ANOVA compared among groups.

**Fig 3:**
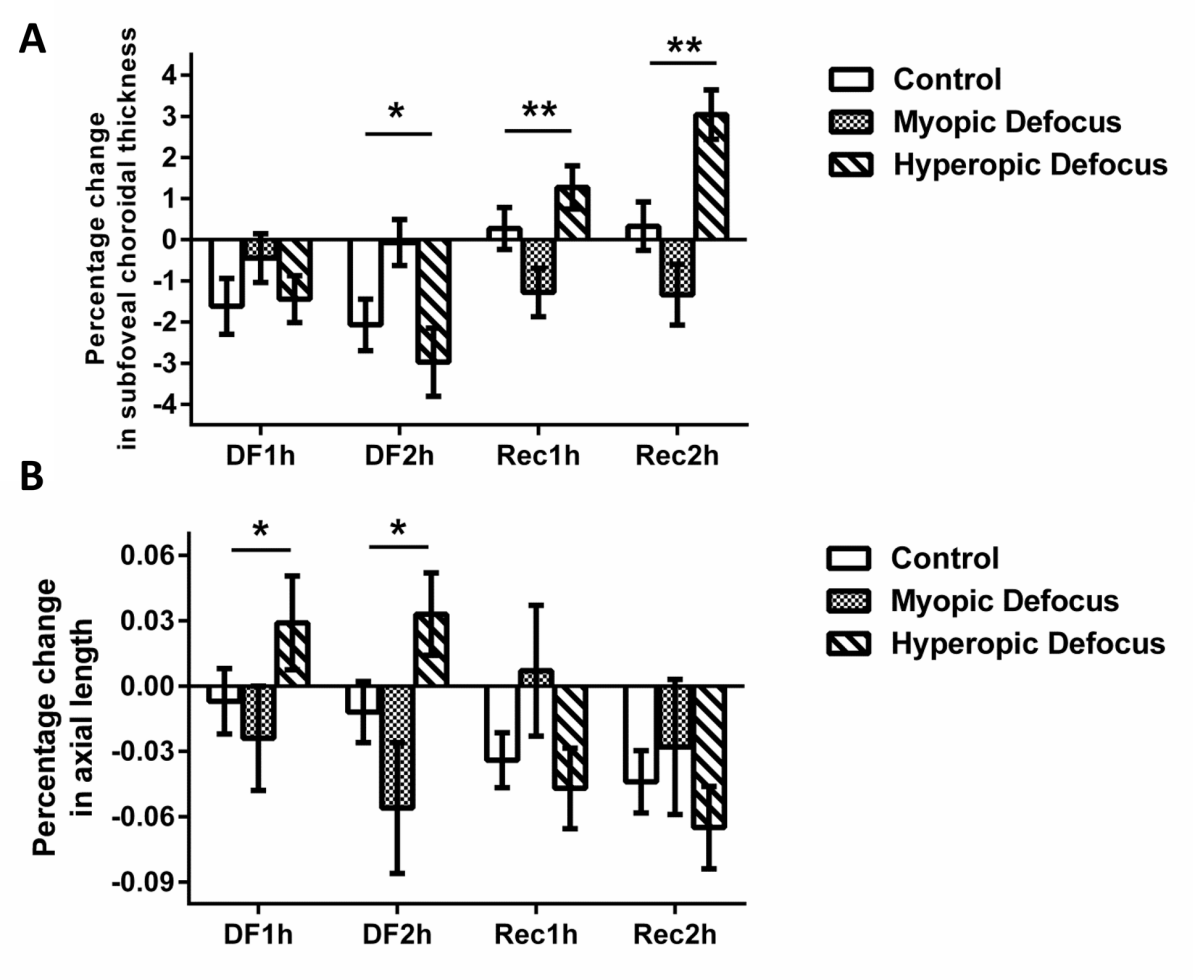
Changes in subfoveal choroidal thickness (SFChT) and axial length (AXL) in response to optical defocus. Percentage change vs. baseline in SFChT (A) and AXL (B) in control group (CG), myopic defocus group (MDG) and hyperopic defocus group (HDG) during the study. DF1h and DF2h denote 1 and 2 hours after receiving optical defocus, respectively. RE1h and RE2h denote 1 and 2 hours after removing the optical defocus, respectively. Data shown are in mean ± SEM, * p < 0.05, ** p < 0.01, two-way ANOVA.

The change in SFChT in the MDG and HDG at 1 hour was not significantly different from the CG. However, at 2 hours, the choroidal thickness was significantly altered (p < 0.05, Fig 3A). A decrease in SFChT was found in the HDG whereas a relatively thickening of subfoveal choroid was observed in in the MDG (mean percentage change at 2-hour defocus; control vs. hyperopic defocus vs. myopic defocus; -2.07 ± 0.63% vs. -2.98 ± 0.83% vs. -0.07 ± 0.56%). The imposed optical defocus was then removed for 2 hours and recovery of choroidal thickness was observed. During the recovery period, the subfoveal choroid significantly thickened in the HDG and thinned in the MDG (p < 0.01). The subfoveal choroid did not change significantly in the control group. The difference in SFChT change was significant after 2 hours of recovery (mean percentage change at 2-hour recovery; control vs. hyperopic defocus vs. myopic defocus; 0.33 ± 0.59% vs. 3.04 ± 0.60% vs. -1.34 ± 0.74%). The magnitude of choroidal thickening in the eyes recovering from hyperopic defocus after 2 hours was greater than that at the 1-hour interval (p < 0.01, paired t-test).

In addition to SFChT, AXL was measured every hour with optical defocus. The AXL exhibited changes in an opposite direction to the choroidal thickness. Significant shortening of AXL was observed in eyes treated with myopic defocus for 1 hour (p < 0.05, Fig 3B) and it was further shortened after 2 hours. In contrast, the AXL was significantly elongated in response to hyperopic defocus. During the 2-hour recovery period, there was no significant difference between the AXL in the control, myopic and hyperopic defocus groups. The left eyes from the MDG, HDG and both eyes from CG received full correction for distance vision over the entire experimental period (4 hours).

Some diurnal changes were noted in the control group. While the change in AXL during the first 2 hours was small and not statistically significant in the control group, AXL decreased in the afternoon, i.e., from 12:00 p.m. to 2:00 p.m. (mean change at 2:00 p.m. in CG ± SEM: -11.25 ± 3.64 μm, repeated measurement, p < 0.05, Table 2). Also, SFChT thinned in the control group in the first 2 hours (mean change at 12:00 p.m. ± SEM: -5.03 ± 1.48 μm, repeated measurement, p < 0.05) and then increased in the afternoon (12:00 p.m. to 2:00 p.m., mean change at 2:00 p.m. ± SEM: 0.28 ± 1.84 μm). The magnitude of changes in both AXL and SFChT are comparable to the previous literatures [22, 26].

**Table 2:** Changes in axial length and subfoveal choroidal thickness (SFChT) in response to control, myopic and hyperopic defocus.

	**Control**		**Myopic defocus**		**Hyperopic defocus**	
Treated eye	**Axial length / μm**	**SFChT / μm**	**Axial length / μm**	**SFChT / μm**	**Axial length / μm**	**SFChT / μm**
**Defocus 1 hr**	-1.76 ± 3.76	-3.89 ± 1.61*	-5.88 ± 6.19	-1.41 ± 1.51	7.65 ± 5.53	-3.54 ± 1.33*
**Defocus 2 hrs**	-2.94 ± 3.61	-5.03 ± 1.48*	-14.12 ± 7.53	-0.36 ± 1.43	8.23 ± 4.79	-6.83 ± 1.89*
**Recovery 1 hr**	-7.65 ± 3.27*	0.64 ± 1.45	1.76 ± 7.54	-3.43 ± 1.30*	-11.76 ± 4.64*	2.25 ± 1.10
**Recovery 2 hrs**	-11.25 ± 3.64*	0.28 ± 1.84	-7.06 ± 7.8	-3.75 ± 1.87	-16.47 ± 4.85*	6.32 ± 1.30†
Control eye						
**Defocus 1 hr**	3.53 ± 7.99	-2.34 ± 1.74	1.76 ± 8.58	-3.49 ± 2.16	-2.35 ± 0.76	0.024 ± 1.04
**Defocus 2 hrs**	5.29 ± 1.99	-5.36 ± 1.75†	-0.59 ± 1.62	-4.87 ± 2.19*	-1.18 ± 1.06	-0.81 ± 1.48
**Recovery 1 hr**	-11.18 ± 0.93*	2.63 ± 1.14*	-21.76 ± 1.23†	3.49 ± 1.49*	0.59 ± 0.82	0.049 ± 1.23
**Recovery 2 hrs**	-14.37 ± 1.27*	2.15 ± 1.9	-14.12 ± 0.83†	3.41 ± 1.57*	-15.88 ± 2.77	2.48 ± 1.60

Changes shown in the first two hours (Defocus 1 hr and 2 hrs) refer to the changes compared to the baseline measurement. Changes shown during the recovery period (Recovery 1 hr and 2 hrs) represents the difference after the defocus treatment.

Data are shown as mean ± SEM

* p < 0.05

† p < 0.01 repeated measure ANOVA.

In order to examine a potential contralateral eye effect in groups wearing defocus, changes in AXL and choroidal thickness of the control eyes (i.e. the left eyes) in all three groups were analyzed. They were not significantly different between three groups (two-way ANOVA, p > 0.05). This indicated that AXL and SFChT of the control eyes from the three groups were not affected by defocus-induced changes occurring in the contralateral eyes. In the left eyes of all three groups AXL was stable in the morning and decreased after noon, while SFChT thinned in the morning and became thicker in the afternoon.

## Discussion

In the current study, short-term exposure to optical defocus led to a small but significant change in AXL and SFChT in Chinese schoolchildren. After two hours of exposure, AXL increased and SFChT decreased in the group with hyperopic defocus. In contrast, AXL decreased and the change in SFChT was minimal but the choroid was relatively thicker than that in control group in response to myopic defocus. To our knowledge, this is the first report to demonstrate a significant change in thickness of choroids in response to optical defocus in children. Similar changes have been observed in avians [2, 6], primates [4] and young adults [22, 26, 27].

The magnitude of choroidal changes found in the current study differ from those observed in previous studies with chicks [2] and young adults [22, 26, 28]. Compared to baseline choroidal thickness of chicks increased by 53 μm after one hour of exposure to +3D myopic defocus [29]. In young emmetropic adults receiving the same amount of myopic defocus for an hour choroidal thickness increased by approximately 12 μm compared to baseline [27]. A recent study on young East Asians even demonstrated a larger magnitude in choroidal thickening (approximately 20 μm to +2D myopic defocus) [28]. Contrary to these findings in young adults, no absolute thickening of SFChT was induced by myopic defocus in the current study. In this study, in eyes with myopic defocus SFChT thinned but significantly less than in the control group (mean change ± SEM at 2 hours; myopic defocus vs. control; -0.36 ± 1.43 μm vs. -5.03 ± 1.48 μm). Although there was no absolute increase in SFChT vs. baseline values in the treated eye, there was significant relative choroidal thickening in response to myopic defocus when the comparison was made against the control group and, thereby, accounted for diurnal changes in SFChT. The magnitude of choroidal thinning experienced in the control group for the first two hours was comparable to diurnal variation found in the previous studies [11, 22]. One may argue that the application of cyclopentolate may diminish the choroidal thickening to myopic defocus. The presence of choroidal thickness changes after mydriatics remains unknown because different studies came to contradictory results [30–32]. It will be optimal if we could measure the choroidal thickness before application of cyclopentolate. Although we could not rule out this possibility, the effect of myopic defocus could still be observed when we compared to the control group in which cyclopentolate was also instilled for eliminating the effect of accommodation on choroidal thickness. Previous studies showed cyclopentolate reaches maximum cycloplegia in 20 to 60 minutes and its effect could last for 6 to 24 hours [33, 34]. Although the duration seems to be long enough for the course of the current study, the accommodation may still have potential effect on choroidal thickness as the amplitude of accommodation resumes gradually after the instillation. Around 30% of accommodative amplitude was returned by 3 hours post instillation [33, 34]. Another possible cause for the discrepancy could be the difference in lighting conditions during the experimental procedures. Data was collected under room lighting (~430 lux) in our study while the previous studies were carried out under relatively dim lighting (~10 lux).

Our results indicate that the magnitude of choroidal changes in the tested children is lower than in chicks and young Caucasian and East Asian adults, especially in response to myopic defocus. We expected to see a more robust change in choroidal thickness in children than in adults in response to myopic defocus. However, the amount of thickening reported in Caucasian and East Asian young adults were much larger than the changes observed in this study with Chinese children. Furthermore, the same amount of hyperopic defocus caused more choroidal thinning in Chinese children than in young Caucasian adults [26] but less when compared to the East Asian adults [28]. The data may suggest the patterns of response to optical defocus differ between Chinese children, Caucasian and East Asian adults with Chinese children being more responsive to hyperopic defocus in terms of choroidal response. While the exact reason for these differences is unknown, race and age are potential factors of relevance. Testing Caucasian children could provide additional information about the relative impact of race and age on choroidal response.

Our results showed the choroidal response to myopic and hyperopic defocus differed quantitatively, despite the same amount of defocus. Greater changes to hyperopic defocus were observed. This is in stark contrast to the choroidal response in chicks where the chick’s choroid is more responsive to myopic defocus even when the same amount of hyperopic and myopic defocus is projected into the eye simultaneously [35]. Therefore, although both human and chick choroids responded to optical defocus in a qualitatively similar manner (thinning and thickening), the responses were very different quantitatively.

The current results also demonstrated the choroidal changes induced by hyperopic or myopic defocus were reversible. The choroid thickened after the cessation of hyperopic defocus whereas it thinned significantly during recovery from myopic defocus. Similar recovery of the choroidal thickness has been demonstrated in previous studies [2, 26] which may act to compensate for the optical defocus by modifying the position of the retinal plane accordingly. Moreover, the changes in choroidal thickness were only present in eyes with optical defocus while the choroids in the contralateral eyes remained unaffected, i.e., their thicknesses were similar to that in the untreated control group. This suggests that the choroidal response to optical defocus is restricted to the treated eye and any cross talk or yoking effect between two eyes is minimal.

Changes in choroidal thickness and axial length to optical defocus are anticipated to be opposite to each other. When the choroid thickens in response to myopic defocus, axial length will become shorter, since the axial length, when measured by IOL Master, is defined as the distance between anterior cornea and RPE. However, this pattern was barely observed during the experimental period. This is likely due to the limited resolution of the axial length measurement with which the minimal detectable difference is 10 μm [36]. Choroidal thickness in children also exhibits a similar pattern of diurnal variation shown in young adults [11, 26]. The choroid thins in the morning and then thickens in the afternoon. The magnitude of the thinning in the morning in the current study was comparable to the previous reports (less than 10 μm). However, we could not compare the total diurnal amplitude among studies because we only monitored the changes for 4 hours while the others measured the choroidal thickness for 8 to 12 hours.

In order to delay or prevent development of myopia, many optical interventions that impose myopic defocus, such as orthokeratology, bifocal and multifocal contact lenses, have been designed. These interventions could inhibit the ocular elongation [18, 19, 37]. However, the efficacy of these optical interventions is ultimately assessed by refractive status and AXL which change slowly and typically require at least three to six-month post treatment for stable results. Some may suggest even twelve-month post treatment is more appropriate when seasonal variation in eye growth is taken into consideration [38]. Since choroidal thickness changes rapidly to optical defocus, it may provide a quick feedback on the retinal response to defocus (especially myopic defocus). Based on the results of the current study, we hypothesize that choroidal thickening may foretell the ocular responses to myopic defocus. Whether the changes in choroidal thickness are proportional to the degree of optical defocus remains unknown. Once a dose relationship between myopic defocus and choroidal thickening is established, the choroidal thickness measured by SD-OCT may act as a novel parameter for clinicians to monitor the effect of myopic defocus during myopic control interventions.

In conclusion, our study showed for the first time that short-term optical defocus in myopic children leads to a change in choroidal thickness. These changes were reversible after removal of optical defocus. Our results suggest that the visual system of children is sensitive to both myopic and hyperopic defocus.

## Supporting Information

S1 File Raw data of subfoveal choroidal thickness measurement. (XLSX)
